# Does Online Community Participation Contribute to Medication Adherence? An Empirical Study of Patients with Chronic Diseases

**DOI:** 10.3390/ijerph18105100

**Published:** 2021-05-12

**Authors:** Jyh-Jeng Wu, Yueh-Mei Chen, Paul C. Talley, Kuang-Ming Kuo

**Affiliations:** 1 Department of Business Management, National United University, Miaoli 360001, Taiwan; jjwu@nuu.edu.tw (J.-J.W.); ymei08@gmail.com (Y.-M.C.); 2 Miaoli General Hospital, Ministry of Health and Welfare, Miaoli 36054, Taiwan; 3 Department of Applied English, I-Shou University, Kaohsiung 84001, Taiwan; atlanta.ga@msa.hinet.net

**Keywords:** emotional support, informational support, medication adherence, online communities, self-efficacy, trust

## Abstract

Effectively improving the medication adherence of patients is crucial. Past studies focused on treatment-related factors, but little attention has been paid to factors concerning human beliefs such as trust or self-efficacy. The purpose of this study is to explore the following aspects of patients with chronic diseases: (1) The relationship between emotional support, informational support, self-efficacy, and trust; (2) the relationship between self-efficacy, trust, and medication adherence; and, (3) whether chronic patients’ participation in different types of online communities brings about significant statistical differences in the relationships between the abovementioned variables. A questionnaire survey was conducted in this study, with 452 valid questionnaires collected from chronic patients previously participating in online community activities. Partial Least Squares-Structural Equation Modeling analysis showed that emotional support and informational support positively predict self-efficacy and trust, respectively, and consequently, self-efficacy and trust positively predict medication adherence. In addition, three relationships including the influence of emotional support on trust, the influence of trust on medication adherence, and the influence of self-efficacy on medication adherence, the types of online communities result in significant statistical differences. Based on the findings, this research suggests healthcare professionals can enhance patients’ self-efficacy in self-care by providing necessary health information via face-to-face or online communities, and assuring patients of demonstrable support. As such, patients’ levels of trust in healthcare professionals can be established, which in turn improves their medication adherence.

## 1. Introduction

Adherence refers to a process in which patients take medication according to medical advice [1]. Successful treatment relies on stable adherence to medical guidance, and medicine is the most important treatment order [2]. Medication adherence can not only alleviate disease symptoms, delay the development of diseases, and reduce the risk of complications but also improve a person’s quality of life [3]. In particular, patients suffering from chronic diseases must take medication on time according to the medical advice given in order to effectively control chronic diseases. If the patients fail to follow medical advice, they may have poor health outcomes and may incur higher medical care costs in the future [4]. Poor medication adherence of patients with chronic diseases is actually a global problem. In 2003, the World Health Organization (WHO) reported that medication adherence of chronic patients in developed countries was barely 50% [5]. Since then, there has been no significant improvement in the problem [2,3], and the medical costs derived from poor medication adherence of chronic patients have continued to rise [6,7]. For example, three conditions that incurred the highest outpatient healthcare costs were all chronic diseases: chronic kidney disease, type 2 diabetes, and hypertension. The total medical expenditure in 2019 incurred due to the three conditions was approximately 273 million USD (approximately 20.6% of total outpatient expenses in 2019), indicating that chronic patients’ poor medication adherence must be addressed properly [8], or the problem will have adverse effects on patients’ health and the availability of overall medical resources.

Recent evidence shows that many authors have discussed the factors affecting medication adherence [1,9,10]. However, the true effects of those influencing factors on medication adherence are yet unconfirmed [1,9]. Furthermore, prior evidence primarily focuses on treatment-related factors [10], while factors concerning human beliefs, such as trust or self-efficacy, remain largely unexamined. In addition, previous research has established that educating patients could improve their medication adherence [11]. Therefore, educating patients by means of online health communities which provide relevant health information has gradually received attention [12]. However, little research concerning the effects of online communities on the medication adherence of chronic patients has been conducted. In order to bridge this knowledge gap, this study attempts to analyze the influencing factors of medication adherence for chronic patients who participate in online health communities based on social cognitive theory and social support. The main objectives of this research include: (1) To identify the relationship between emotional support, informational support, self-efficacy, and trust of chronic patients; (2) to identify the relationship between self-efficacy, trust, and medication adherence of chronic patients; and, (3) to analyze whether chronic patients’ participation in professional online communities brings about significant statistical differences in the relationships between the abovementioned variables.

## 2. Related Work

### 2.1. Social Support

Social support refers to the psychological or material support provided by others to assist individuals in responding to problems or stress [13,14]. Social support is a widespread phenomenon that covers social networks and social integration. It is usually generated as part of the communication between individuals, and the degree of social support that an individual receives is often measured by the nature of social relationships. Social relationships are generally divided into two categories: structural and functional. A structure-based relationship is also known as social integration, which refers to the degree of social network connection formed by an individual with members of the community and family. A function-based relationship refers to the support that is accepted or perceived by a member of public through the relationship partners; that is, in the social relationship structure, social network members can provide specific functions, such as emotions, information, or substantial support [13,15]. Prior evidence [13,15] categorized functional-level support into: (1) cognitive support—others provide the desired support in times of need; (2) substantial support—others provide support in terms of finance, materials, or services; (3) emotional support—expressions of comfort, care, concern, and encouragement, (4) sense of belonging—social activities are shared by peers or the sense of belonging to the society is provided by a certain person; (5) tangible support—support from family members; and (6) informational support—others provide useful advice or guidance.

The main purpose of this study is to explore the factors influencing the medication adherence of chronically ill populations. Medication adherence is closely related to having close friends or the ability of medical teams to provide individual patient support [1,10]. These people can provide emotional and informational support (e.g., medication reminders and medication guidance) continuously to meet patients’ needs [16]. This study defines emotional support as the degree to which chronic patients receive care, encouragement, or comfort from others related to their health status [15,17], while informational support is defined as the extent to which chronic patients receive relevant information such as advice and guidance from others relating to medication [15].

### 2.2. Social Cognitive Theory

Social cognitive theory holds that individuals’ actions and behaviors are learned by observing others’ behavior in the society. Through self-motivation and self-regulation mechanisms, the learning of personal knowledge and behavior can be formed, and these learning behaviors have a strong influence on an individual’s behavior [18]. Such observational learning and social experiences mainly generate effects through the concept of self-efficacy during personality development [19]. Self-efficacy mainly refers to the strength of one’s belief in one’s own ability to accomplish tasks and achieve goals [19]. Previous studies have found that a strong sense of self-efficacy is related to better social integration, while a low sense of self-efficacy is related to depression, anxiety, and helplessness. A strong cognition of self-efficacy can also improve an individual’s cognitive processes and performance in a given environment, including decision quality, goal setting, and achievement [19,20]. Self-efficacy can also be used to explain and predict psychological changes, therapeutic effects, and behaviors after receiving different clinical treatments [19]. Adherence to medical advice is included [21]. This study defines self-efficacy as the extent to which chronic patients have sufficient capacity to take care of their own health needs.

### 2.3. Trust

Trust is the key to maintaining positive interpersonal relationships in an environment [22], and it is at the core of interactions between people [23]. Cook and Wall [24] define trust as the extent to which one is willing to ascribe good intentions to others and to have confidence in their words and actions. Trust is also considered as a form of dependency [25]. Trust is an essential element in what actions people choose to take. When there is mutual trust between people, values and norms can be constructed with trusted individuals or groups to achieve common commitments [26,27,28,29]. McKnight and Chervany [30] argued that one would first develop a belief in trust, then an intention to trust, and finally a behavior of trust. In the context of this study, since trust is considered as a crucial precursor to cooperation, when a patient with a chronic disease believes that a physician can offer treatment (trust belief), the acceptance intention is based on the degree of trust (trust intention), and finally, the patient develops a behavior to cooperate with the physician (trust behavior), i.e., follows the physician’s medical advice. Previous literature has clarified that cooperation with a physician is difficult to realize without trust being present [31]. This study defines trust as the degree to which chronic patients trust healthcare professionals to meet their individual needs.

### 2.4. Medication Adherence

Medication adherence is often considered as a vital solution to control chronic disease [21], but the thoughts, feelings, habits, behaviors, and preferences of chronic patients are often considered as barriers to medication adherence [10,32]. This may be due in part to patients’ concerns about potential adverse effects with the medication, drug dependency, or the complexity of the medication regime [33,34]. Prior studies [1,10] roughly categorized the factors that influence medication adherence into patient demographic factors (e.g., age or education level); treatment-related factors (e.g., dosage or frequency of medication); patients’ health condition factors (e.g., self-consciousness about one’s health); and, patients’ socioeconomic status factors (e.g., social support or emotional support). However, the effects of quite a few of these factors on medication adherence have yet to be confirmed [1,9], and a significant portion of the literature is focused on treatment-related factors [10], which may lead to a possible neglect of important factors related to human beliefs such as personal trust or self-efficacy. In addition, meta-analysis by Kini and Ho [11] shows that patient education is also an effective way to improve patients’ medication adherence. Given the rapid development of the Internet and information technology, more and more online communities are being set up to provide health information to educate patients [12]. However, few studies analyze the effect of online community participation on the medication adherence for chronic patients. This study defines medication adherence as the degree to which chronic patients can actively follow the physicians’ recommendations regarding prescribed and over-the-counter medication.

### 2.5. Research Model and Hypotheses

**Figure 1:**
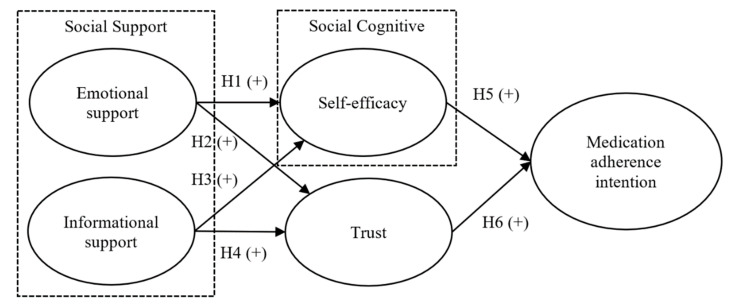
Research model (+ means positive direction).

This study integrates social support perspective, social cognitive theory, and trust, to investigate the factors influencing the possible levels of medication adherence of chronic patients. From the perspective of social support, when chronic patients obtain sufficient emotional support and disease-related informational support, they will not only acquire requisite knowledge about chronic diseases but also develop self-motivational and self-regulatory behaviors, which, as a result, enables them to learn how to deal with their individual conditions. Furthermore, trust in healthcare professionals can be nurtured by interactions such as self-confidence in one’s capacity to comply with medical advice and trust in the medical team, which are both expected. This, in turn, enhances the willingness of chronically ill patients to follow medical advice concerning their medications. Based on the above inferences, the research model of this study is proposed as Figure 1. Emotional support and informational support are used to predict self-efficacy and trust respectively, while self-efficacy and trust are further expected to predict the medication adherence intention of chronic patients. Accordingly, the relevant hypotheses of this study are derived.

### 2.6. Effect of Emotional Support on Self-Efficacy and Trust

Self-efficacy is defined as confidence in one’s ability to self-regulate one’s own behavior [19]. Previous evidence demonstrates that support from family or friends is positively associated with health self-efficacy [35], while support from friends or family is more important for self-management of chronic diseases [36]. Showing emotional support is an important element of any successful relationship, and it is mainly shown by expressing messages related to care. Such emotional support is the feeling or behavior resulting from intimate relationship support [37]. Without emotional support from friends or family, self-regulation can be hard to achieve. Previous studies have shown that high levels of support from others are beneficial for establishing stable self-regulation behavior, which in turn improves the health of chronic patients [38]. Jackson, Tucker and Herman [35] also showed that social support recognized by an individual is positively linked to one’s health self-efficacy. In terms of trust, Rempel, Holmes and Zanna [39] argued that trust is an important component of intimate relationship. Individuals in stable and trusting relationships tend to communicate with care to satisfy each other’s need for emotional support, while trust can arise when partners respond in a caring or supportive manner [39]. In the field of communication, Weber, Johnson and Corrigan [37] found that the amount of partners’ emotional support felt by the subjects positively was related to their trust in partners. Based on the above discussion, the following hypotheses are proposed in this study:

**Hypothesis** **1** **(H1).**  *Emotional support has a positive effect on the self-efficacy of chronic patients.*

**Hypothesis** **2** **(H2).**  *Emotional support has a positive effect on the trust of chronic patients on healthcare professionals.*

### 2.7. Effect of Informational Support on Self-Efficacy and Trust

Informational support refers to support, in the form of information, which can provide advice or guidance to an individual or is useful to the individual. Such support can assist individuals to solve problems and thus make better decisions [40]. Others who provide informational support may seek information from healthcare professionals or online community websites. These can act as information bridges that help patients to find information from other sources or to share information in online communities [41]. This information may have an impact on chronic patients’ healthcare self-efficacy and their trust in healthcare professionals. Previous literature pointed out that social support can help people cope better, when they are provided with useful resources related to the disease, especially when patients regard medical team support as the most important part of disease self-management [9,38]. Macabasco-O’Connell, DeWalt, Broucksou, Hawk, Baker, Schillinger, Ruo, Bibbins-Domingo, Holmes, Erman, et al. [42] found that when individuals with heart disease are more knowledgeable about the disease, their self-efficacy increases. That is, if individuals can obtain sufficient disease-related information, they will have more confidence in their capacity for self-care. Zhou, Kankanhalli, Yang and Lei [43] demonstrated that if people have sufficient health-related information, they might be willing to interact with healthcare professionals further and become better qualified to judge whether they can trust healthcare professionals’ advisories. Ommen, Janssen, Neugebauer, Bouillon, Rehm, Rangger, Erli and Pfaff [44] also confirmed that informational support provided by physicians is positively and significantly associated with patient’s trust in physicians. Based on the above literature and discussion, the following hypotheses are postulated:

**Hypothesis** **3** **(H3).**  *Informational support has a positive effect on the self-efficacy of chronic patients.*

**Hypothesis** **4** **(H4).**  *Informational support has a positive effect on the trust of chronic patients on healthcare professionals.*

### 2.8. Effect of Self-Efficacy on Medication Adherence Intention

According to social cognitive theory, the concept of self-efficacy holds that for a person to maintain specific behaviors, the main motivation is to control and protect oneself from problematic results [19], while self-regulation is key to the development of self-efficacy. With self-efficacy, patients can maintain specific health behaviors for a long time [36,45], and prior evidence also shows that self-efficacy is an important factor in changing health behaviors [46,47]. Previous studies found that the reasons for non-adherence to medication in patients include concerns about the potential adverse effects of the medication, drug dependence, or the complexity of the medication [33,34]. The solution to these problems is to educate patients on how to control the disease and how to deal with any adverse effects of the medication, thereby facilitating patients’ belief in the need to keep healthy behaviors [48]. Previous literature also demonstrated a positive correlation between self-efficacy and health behaviors. For example, Chen, Yehle, Albert, Ferraro, Mason, Murawski and Plake [49] found that the self-efficacy of heart disease patients is positively associated with self-care. Similarly, Jackson, Tucker and Herman [35] found that health self-efficacy is positively linked to healthy lifestyles. Based on the above literature and discussion, this research postulates the following hypothesis:

**Hypothesis** **5** **(H5).**  *Self-efficacy has a positive effect on the medication adherence of chronic patients.*

### 2.9. Effect of Trust on Medication Adherence Intention

Trust has been widely discussed in various fields. In the medical field, the main reasons patients trust healthcare professionals are that: (1) They believe healthcare professionals are honest and possess satisfactory professional capabilities; and (2) they will consider the best interests of the patient while protecting the privacy of the people [50]. Trust in healthcare professionals may affect people’s willingness to seek medical assistance and disclose sensitive information, and even their willingness to follow medical advice [51]. Therefore, trust plays an essential role in how patients make medical-related decisions. Previous studies confirmed that patients’ trust in physicians is associated with good health outcomes, as patients are more likely to follow medical advice [52,53]. Other evidence further showed that trust and healthy behaviors are positively related [54,55], that is, trust can predict health behaviors [56]. Considering our research context, if chronic patients can trust healthcare professionals, they are more likely to follow medical advice concerning their medication. Based on the aforementioned literature and discussion, this research proposes the following hypothesis:

**Hypothesis** **6** **(H6).**  *Trust has a positive effect on the medication adherence intention of chronic patients.*

## 3. Materials and Methods

### 3.1. Measures

The questionnaire items of this research are designed mainly with reference to relevant literature, and modified according to the research context. This study defines emotional support as the degree to which chronic patients receive care, encouragement, or comfort relating to their health conditions from others. Three items adapted from Liang, Ho, Li and Turban [57] were used to measure emotional support. Informational support is operationalized as the extent that chronic patients receive relevant information such as advice and guidance on mediation from others. Three items adapted from Liang, Ho, Li and Turban [57] are used to measure informational support. Self-efficacy is defined as the degree to which chronic patients have sufficient ability to carry out self-healthcare. Three items are designed for the measurement, mainly adapted from Karademas [58]. Trust is defined as the extent to which chronic patients trust healthcare professionals and three items are adapted from Chow and Chan [59] for the measurement. Medication adherence is operationalized as the degree to which chronic patients can actively follow recommendations of medication. Three items are designed for the measurement, mainly adapted from Fernandez, Chaplin, Schoenthaler and Ogedegbe [60]. Questionnaire items were measured on a 5-point Likert scale, ranging from 1, representing “strongly disagree”, to 5, representing “strongly agree.” Before the formal survey, the first draft of the questionnaire went through a pilot with 91 samples. According to the result of the pilot trial, the wordings of the questionnaire items were modified accordingly. The full questionnaire is given in the Appendix A.

### 3.2. Sample and Investigation Procedure

The main purpose of this study is to explore the factors affecting the medication adherence of chronic patients. The subjects of the survey are chronic patients who have taken at least one prescription medication for a chronic disease and have participated in health-related online communities. Paper questionnaires were distributed for this study between January and March, 2020. The distribution locations included National Health Insurance (NHI) contracted pharmacies. First, the person in charge of the NHI contracted pharmacies was asked to assist with the conduct of the survey. Respondents were then recruited by researchers in the waiting areas or medicine pick-up areas of the NHI contracted pharmacies. The researchers first confirmed whether a respondent met the inclusion criteria. If these criteria were met, they explained the research purpose, content of the questionnaire, and survey method to the respondent. If the respondent agreed, the questionnaire was administered to the patient. The researchers then confirmed the survey’s completeness, filling in any omitted respondent portions on the spot.

The questionnaire of the study was answered anonymously, and convenience-sampling method was adopted. The number of questionnaires distributed was mainly determined according to the population distribution of counties and cities in 2019 disclosed by the Department of Household Registration, Ministry of the Interior of the Republic of China. Approximately 40% of the questionnaires were distributed in Northern Taiwan (Keelung County, Taipei City, New Taipei City, Taoyuan City, and Miaoli County), 29% in Central Taiwan (Taichung City, Changhua County, Nantou County, and Chiayi County), 28% in Southern Taiwan (Tainan City, Kaohsiung City, and Pingtung County), and 28% in Eastern Taiwan (Yilan County, Hualien County, and Taitung County). The research process of this study has been reviewed and approved by the Institutional Review Board of Jianan Psychiatric Center, Ministry of Health and Welfare (No. 19-048).

## 4. Results

### 4.1. Descriptive Statistics

A total of 452 valid questionnaires were collected in this study. The sample comprised 229 men (approximately 50.66%) and 223 women (approximately 49.34%). The majority of the respondents was aged between 50 and 69 (approximately 61.28%). The majority of the respondents (approximately 84.74%) had junior high school, senior high school, or college/university education. The distribution of living areas of respondents was similar to the distribution of the population in Taiwan (see Table 1). All respondents participated in health-related online communities, among which 206 people belonged to healthcare professional communities (approximately 45.58%), and the rest belonged to non-healthcare professional communities.

### 4.2. Model Validation

Partial Least Squares-Structural Equation Modeling was used for data analysis in this study, and the analysis consisted of two stages: the measurement model and the structural model. The measurement model was mainly used to evaluate the reliability and validity of the research items and constructs, while the structural model was used to verify the hypotheses [61].

### 4.3. Measurement Model

In this study, reliability was evaluated by the cross loading of items and composite reliability, while the validity was evaluated by means of convergence validity and discriminant validity. In terms of reliability evaluation, the cross loadings of items in all constructs are higher than the recommended value of 0.7 (as shown in Table 2). Table 3 also shows that the composite reliabilities of all constructs are also higher than the recommended value of 0.7 [61], showing that the items and constructs of this study have sufficient reliability. In terms of validity, Fornell and Larcker [62] suggested that an average variance extraction higher than 0.5 indicates convergence validity. The average variance extractions of all constructs in this study comply with the standard. As for the evaluation of discriminant validity, the correlation coefficient between two different constructs should be less than the square root of the average variation extraction of each construct. Table 3 shows that all constructs in this study have sufficient discriminant validity. According to the abovementioned analysis, the reliability and validity of the items and constructs of this study are both sufficient.

### 4.4. Structural Model

**Figure 2:**
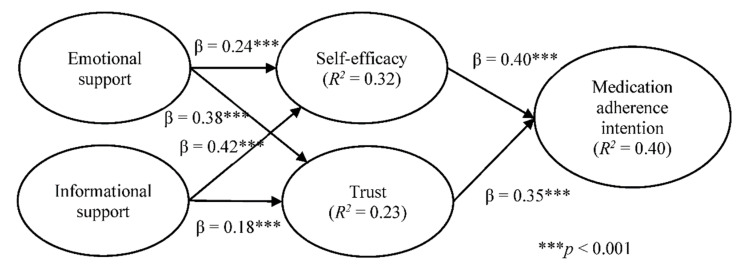
Structural model results.

Structural model results (as shown in Figure 2) indicate that both emotional support and informational support have positive and significant effects on self-efficacy and trust, respectively. Hypotheses H1, H2, H3, and H4 are thus all supported. Self-efficacy and trust also show positive and significant effects on medication adherence; therefore, hypotheses H5 and H6 are also supported. In terms of variance explained in the proposed model, emotional support and informational support jointly explain approximately 32% and 23% of the variance of self-efficacy and trust, respectively. Meanwhile, self-efficacy and trust collectively explain approximately 40% of the variance of medication adherence intention.

### 4.5. The Moderating Effect of Healthcare Professional Community Participation

Multi-group analysis can be used to test if pre-defined groups have statistical differences in the relationships of different variables, or to explore whether variables of different groups have moderating effects [63]. In this study, healthcare professional communities refer to online communities established by hospitals or healthcare professionals that provide specialized medical knowledge. To understand whether participation in different types of online communities have different effects on medication adherence for chronic patients, the communities that the respondents participate in were divided into healthcare professional communities and non-healthcare professional communities. By multi-group analysis, comparisons were made to assess whether there were statistical differences between the two groups of samples in the six hypotheses proposed by this study. We adopted the approach suggested by Sarstedt, Henseler and Ringle [63] by inspecting whether the path coefficient for one group falls within the corresponding confidence interval of another group and vice versa. If there exists no overlap, we can assume that the group-specific path coefficients are significantly different.

**Figure 3:**
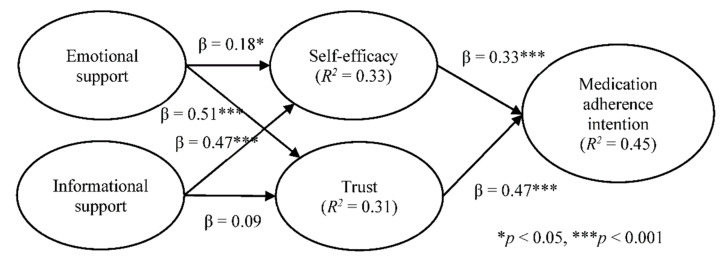
Structural model results for healthcare professional communities.

**Figure 4:**
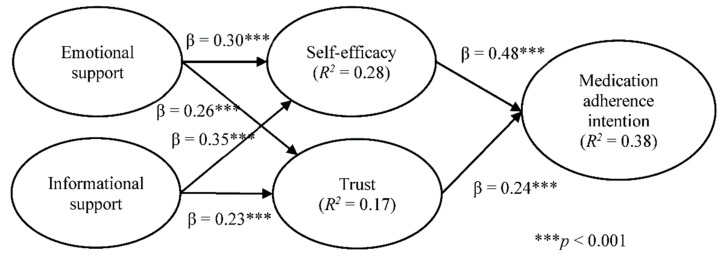
Structural model results for non-healthcare professional communities.

As Table 4 shows, three out of the six hypotheses (i.e., emotional support and trust, self-efficacy and medication adherence, and trust and medication adherence) showed significant statistical differences. Emotional support positively influences trust more strongly for respondents who participated in healthcare professional communities than for those participated in non-healthcare professional communities. Further, self-efficacy and medication adherence had a stronger association for respondents who participated in non-healthcare professional communities than those who participated in healthcare professional communities. Finally, trust had a stronger association with medication adherence for respondents participated in healthcare professional communities than those participated in non-healthcare professional communities. Figure 3 shows the structural model results of healthcare professional communities and Figure 4 for non-healthcare professional communities.

## 5. Discussion

Based on the perspectives of social support and social cognitive theory, this research explores the influencing factors for the medication adherence intentions of chronic patients. Emotional support and informational support were identified as vital factors of self-efficacy and trust, respectively, and self-efficacy and trust were found to positively affect medication adherence. In addition, multi-group analysis showed that participation in health-related online communities had moderating effects on different influencing factors of medication adherence.

Based on our findings, emotional support and informational support have positive effects on self-efficacy, which, in turn, affects medication adherence. This finding is consistent with the findings of Jackson, Tucker and Herman [35] and Macabasco-O’Connell, DeWalt, Broucksou, Hawk, Baker, Schillinger, Ruo, Bibbins-Domingo, Holmes, Erman, et al. [42]. In the present study, informational support was found to have a greater impact on self-efficacy than emotional support. The plausible explanation may be that self-care in situations of chronic diseases requires more professional health information, resulting in informational support having a stronger effect on self-efficacy. Previous studies have also found that informational support from physicians and medical teams has the strongest effect on the self-efficacy of chronic patients [38], which is consistent with the findings of this study.

Both emotional support and informational support were found to be positively correlated to trust, a result in accordance with prior evidence [37,44]. Ommen, Janssen, Neugebauer, Bouillon, Rehm, Rangger, Erli and Pfaff [44] however, found emotional support had a stronger effect on trust than informational support, which is contrary to this study’s finding that the effect of informational support was stronger than that of emotional support. One possible explanation may be that the subjects of the previous research were severely injured patients suffering from acute illness, while the subjects of this study were patients with chronic diseases who need to connect with healthcare professionals on a long-term basis. Therefore, emotional support could, depending on the circumstances, have a higher effect on trust than informational support.

For medication adherence, self-efficacy and trust were both positively associated with medication adherence, a finding consistent with previous evidence [35,49,54,55]. Self-efficacy has a stronger effect on medication adherence than trust. This might be explained by the fact that the realization of medication adherence requires the earnest commitment on the part of chronic patients, and cannot be achieved solely based on trust in healthcare professionals.

For the moderating effects on the research hypotheses brought by whether the online communities joined by the patients are of a healthcare professional nature, the result of multi-group analysis shows that the path coefficients of the two hypothetical paths, namely emotional support on trust and trust on medication adherence, are significantly higher for patients who participate in healthcare professional communities than those who participate in non-healthcare professional communities. Generally speaking, when people participate in healthcare professional communities, apart from personal interests, they may seek to obtain necessary medical information from the communities and have more interactions with healthcare professionals, as they are aware that their professional knowledge of medicine is insufficient [12]. In this study, healthcare professional communities are defined as the communities established by the hospitals or healthcare professionals who are highly professional in the treatment of chronic patients. Such communities can enhance patients’ trust in healthcare professionals, and through such interactions, patients can also feel the emotional support from healthcare professionals. Because an emotional bond is an essential part of building patients’ trust in healthcare professionals [52], the strength of the effect of trust on medication adherence is higher than that of the patients who participate in non-healthcare professional communities. For the patients who participate in non-healthcare professional communities, the path coefficient of self-efficacy on medication adherence is significantly higher than that of the patients who participate in healthcare professional communities. One possible reason inferred by this study is that the patients who participate in non-healthcare professional communities believe they have higher self-efficacy, and thus do not need to participate in healthcare professional communities. They are also able to implement medication adherence in a better manner; therefore, the path coefficient is significantly higher than that of the patients who participate in healthcare professional communities.

Medication adherence is an indispensable and crucial part of effective patient care and achieving clinical treatment goals [64]. Previous research [65] pointed out that a patient-centered care model is composed of both informational support and emotional support. Glasgow, Strycker, Toobert and Eakin [38] also revealed that support from medical teams is a key factor determining whether the patients can follow the medical advice. Spanjol, Cui, Nakata, Sharp, Crawford, Xiao and Watson-Manheim [66] further conceptualize adherence of chronic disease treatment as a co-produced service that requires the participation of healthcare professionals and patients so that the value of the service can be revealed. As healthcare professionals are the most trusted group from the perspective of patients [67], apart for providing treatment and medication information to the patient, they have a responsibility to educate patients about how to control diseases with medication and provide information related to medication, such as what and how adverse effects of medication may occur and under what conditions and when patients can reduce the use of drugs safely. Healthcare professionals should also ensure patients feel that they be being cared for and supported. By understanding patients’ usage of drugs more and correcting them without blaming, not only can patients increase their self-care efficacy but also their trust in medical care professionals will be strengthened, which will finally improve their medication adherence.

Concerning the moderating effect of whether the online communities joined by patients are of a healthcare professional nature, as the process of clinical care is restricted by the number of patients waiting to be cared for and time, healthcare professionals may not be able to interact with patients for long periods. This study suggests that apart from regular care sessions at work, healthcare professionals should also try to interact with patients through online websites relevant to the health professions. In addition to providing necessary and accurate health information, the process of interaction allows patients to feel that they are being supported. In such ways, the patients’ self-efficacy in adhering to medical guidelines can be enhanced, and at the same time, the patients’ trust in healthcare professionals can be built, which in turn improves the medication adherence of patients.

Convenience sampling was adopted in this study and the samples do not cover all counties and cities of Taiwan. Therefore, the samples may not be able to represent the opinions of all groups of chronic patients, and the extrapolations of the research results may be restricted. The scope of sample collection can be expanded in future studies. In addition, this study is a cross-sectional study, which only focuses on observing a single point in time. Future studies may consider using longitudinal research methods to understand the effect of time changes on the relationships between variables in this study. Finally, the elderly population, who may be the biggest offenders in terms of adherence to consistent medication usage, may not embrace the use of the Internet as readily and easily as the younger population. Future research should determine how to further motivate the elderly population to actively engage in beneficial healthcare-related virtual communities in order to improve their medication adherence habituation.

## 6. Conclusions

While medication non-adherence remains a problem, it is not an unattainable one. Our findings will be useful to healthcare facilities to foster effective programs to improve non-adherence to medication of chronic patients. First of all, apart for providing treatment and medication information to the patient, healthcare professionals have a responsibility to educate patients about how to control diseases with medication and provide information related to medication. Second, healthcare professionals should also ensure that patients feel that they are being cared for and supported. Last but not least, apart from regular care sessions at work, healthcare professionals should also try to interact with patients through online websites relevant to the health professions. In such ways, the patients’ self-efficacy in adhering to medical guidelines can be enhanced, and at the same time, the patients’ trust in healthcare professionals can be built, which in turn improves the medication adherence of patients.

## Figures and Tables

**Table 1:** Descriptive statistics.

Characteristics		Frequency	Percentage
Gender	Male	229	50.66
	Female	223	49.34
Age	≤39	27	5.97
	40–49	88	19.47
	50–59	121	26.77
	60–69	156	34.51
	70–79	54	11.95
	≥80	6	1.33
Education	Elementary school	39	8.63
	Junior high school	111	24.56
	Senior high school	143	31.64
	College/University	129	28.54
	Graduate school	30	6.64
Living area in Taiwan	North	246	54.42
	Middle	76	16.81
	South	125	27.65
	East	5	1.11
Type of online social community participation	Healthcare	206	45.58
	Non-healthcare	246	54.42

**Table 2:** Cross loading.

Item/Construct	Emotional Support	Informational Support	Self-Efficacy	Trust	Medication Adherence
Emotion1	**0.857**	0.325	0.306	0.359	0.488
Emotion2	**0.913**	0.386	0.365	0.417	0.461
Emotion3	**0.904**	0.437	0.440	0.432	0.452
Information1	0.462	**0.840**	0.433	0.313	0.355
Information2	0.307	**0.878**	0.422	0.249	0.280
Information3	0.336	**0.839**	0.475	0.306	0.355
Self-efficacy1	0.401	0.477	**0.919**	0.375	0.533
Self-efficacy2	0.387	0.491	**0.892**	0.390	0.476
Self-efficacy3	0.355	0.451	**0.907**	0.378	0.484
Trust1	0.378	0.288	0.340	**0.821**	0.419
Trust2	0.340	0.236	0.319	**0.790**	0.415
Trust3	0.406	0.318	0.380	**0.866**	0.453
Adherence1	0.472	0.395	0.532	0.422	**0.832**
Adherence2	0.415	0.324	0.426	0.426	**0.815**
Adherence3	0.361	0.202	0.351	0.409	**0.766**

**Table 3:** Reliability and validity.

Construct	M	SD	CR	AVE	A	B	C	D	E
Emotional Support (A)	4.020	0.727	0.921	0.795	**0.892**				
Informational Support (B)	3.800	0.733	0.889	0.727	0.434	**0.853**			
Self-efficacy (C)	3.670	0.725	0.932	0.821	0.421	0.522	**0.906**		
Trust (D)	4.240	0.549	0.866	0.683	0.454	0.342	0.420	**0.826**	
Medication Adherence (E)	3.970	0.525	0.847	0.647	0.521	0.390	0.550	0.520	**0.805**

Note: M denotes mean, SD denotes standard deviation, CR denotes composite reliability, and AVE denotes average variance extracted.

**Table 4:** Multi-group analysis of online community participation.

Path	β_Yes_	95% Confidence Interval	β_No_	95% Confidence Interval	Significant Difference at 0.05
		Group_No_		Group_Yes_	
Emotional support→Self-efficacy	0.178	[0.167, 0.432]	0.301	[0.021, 0.329]	No
Emotional support→Trust	0.470	[0.227, 0.469]	0.348	[0.340, 0.604]	No
Informational support→Self-efficacy	0.510	[0.135, 0.386]	0.261	[0.356, 0.669]	Yes
Informational support→Trust	0.087	[0.097, 0.365]	0.232	[−0.080, 0.244]	No
Self-efficacy→Medication adherence intention	0.332	[0.368, 0.580]	0.478	[0.201, 0.453]	Yes
Trust→ Medication adherence intention	0.467	[0.135, 0.336]	0.236	[0.318, 0.624]	Yes

Note: β_Yes_ denotes path coefficient of healthcare professional community group, β_No_ denotes path coefficient of non-healthcare professional community group, Group_Yes_ means healthcare professional community group, and Group_No_ means non-healthcare professional community group.

## Data Availability

The data used to support the findings of this study are available from the corresponding author upon reasonable request.

## Appendix A

**Table A1:** Questionnaire Items.

Constructs (Source)	Items
Emotional support [57]	When I have a health problem, my family or friends care for me.
	When I have a health problem, medical staff will give me comfort and encouragement.
	Medical staff are always concerned about my health.
Informational support [57]	When I take a chronic disease medication, the community companion will give me advice.
	When I take prescribed medication, I will look for information on the Internet.
	When I take chronic medications, I will look for information on pertinent websites.
Self-efficacy [58]	I am positive about my health care and have adjustment plans.
	I set my health goals.
	Most of the time, I stick to my health plan.
Trust [59]	I can talk to my medical staff about my health problems very comfortably.
	When I have doubts about medication, the medical staff will respond to me appropriately.
	I understand the medication information given to me by medical staff.
Medication adherence [60]	In order to treat or control chronic diseases to maintain health, I will work on health promotion and take medication on time every day.
	Mostly, I can take my medicine on time.
	I will follow the doctor’s instructions to take the medicine.

## References

[1] Kardas P., Lewek P., Matyjaszczyk M. (2013). Determinants of patient adherence: A review of systematic reviews. Front. Pharmacol..

[2] Zullig L.L., Granger B.B., Bosworth H.B. (2016). A renewed Medication Adherence Alliance call to action: Harnessing momentum to address medication nonadherence in the United States. Patient Prefer. Adherence.

[3] Brown M.T., Bussell J.K. (2011). Medication Adherence: WHO Cares?. Mayo Clin. Proc..

[4] Phillips L.A., Leventhal H., Leventhal E.A. (2013). Assessing theoretical predictors of long-term medication adherence: Patients’ treatment-related beliefs, experiential feedback and habit development. Psychol. Health.

[5] World Health Organization (2003). Adherence to Log-Term Therapies: Evidence for Action.

[6] Lloyd J.T., Maresh S., Powers C.A., Shrank W.H., Alley D.E. (2019). How Much Does Medication Nonadherence Cost the Medicare Fee-for-Service Program?. Med. Care.

[7] Nasseh K., Frazee S.G., Visaria J., Vlahiotis A., Tian Y. (2012). Cost of medication nonadherence associated with diabetes, hypertension, and dyslipidemia. Am. J. Pharm. Benefits.

[8] Ministry of Health and Welfare 2019 Annual Report of National Health Insurance. Proceedings of the Taipai City Ministry of Health and Welfare.

[9] Gast A., Mathes T. (2019). Medication adherence influencing factors—An (updated) overview of systematic reviews. Syst. Rev..

[10] Shahin W., Kennedy G.A., Stupans I. (2019). The impact of personal and cultural beliefs on medication adherence of patients with chronic illnesses: A systematic review. Patient Prefer. Adherence.

[11] Kini V., Ho P.M. (2018). Interventions to Improve Medication Adherence: A Review. JAMA.

[12] Hagg E., Dahinten V.S., Currie L.M. (2018). The emerging use of social media for health-related purposes in low and middle-income countries: A scoping review. Int. J. Med. Inform..

[13] Cohen S. (2004). Social Relationships and Health. Am. Psychol..

[14] Thoits P.A. (2011). Mechanisms Linking Social Ties and Support to Physical and Mental Health. J. Health Soc. Behav..

[15] Holt-Lunstad J., Uchino B.N., Glanz K., Rimer B.K., Viswanath K. (2015). Social Support and Health. Health Behavior: Theory, Research, and Practice.

[16] Scheurer D., Choudhry N., Swanton K.A., Matlin O., Shrank W. (2012). Association between different types of social support and medication adherence. Am. J. Manag. Care.

[17] Langford C.P.H., Bowsher J., Maloney J.P., Lillis P.P. (1997). Social support: A conceptual analysis. J. Adv. Nurs..

[18] Bandura A. (2001). Social Cognitive Theory: An Agentic Perspective. Annu. Rev. Psychol..

[19] Bandura A. (1977). Self-efficacy: Toward a unifying theory of behavioral change. Psychol. Rev..

[20] Maddux J.E. (1995). Self-Efficacy, Adaptation, and Adjustment: THEORY, Research, and Application.

[21] DiMatteo M.R., Giordani P.J., Lepper H.S., Croghan T.W. (2002). Patient adherence and medical treatment outcomes a meta-analysis. Med. Care.

[22] Lewis J.D., Weigert A. (1985). Trust as a social reality. Soc. Forces.

[23] Berscheid E. (1994). Interpersonal relationships. Annu. Rev. Psychol..

[24] Cook J., Wall T. (1980). New work attitude measures of trust, organizational commitment and personal need non-fulfilment. J. Occup. Psychol..

[25] Mayer R.C., Davis J.H., Schoorman F.D. (1995). An Integrative Model of Organizational Trust. Acad. Manag. Rev..

[26] Hsu M.-H., Chang C.-M. (2014). Examining interpersonal trust as a facilitator and uncertainty as an inhibitor of intra-organisational knowledge sharing. Inf. Syst. J..

[27] Hsu M.-H., Chang C.-M., Yen C.-H. (2011). Exploring the antecedents of trust in virtual communities. Behav. Inf. Technol..

[28] Olivero N., Lunt P. (2004). Privacy versus willingness to disclose in e-commerce exchanges: The effect of risk awareness on the relative role of trust and control. J. Econ. Psychol..

[29] Yoon S.-J. (2002). The antecedents and consequences of trust in online-purchase decisions. J. Interact. Mark..

[30] McKnight D.H., Chervany N.L. (1996). The Meanings of Trust.

[31] Dibben M.R., Morris S.E., Lean M.E.J. (2000). Situational trust and co-operative partnerships between physicians and their patients: A theoretical explanation transferable from business practice. Qjm Int. J. Med..

[32] Pound P., Britten N., Morgan M., Yardley L., Pope C., Daker-White G., Campbell R. (2005). Resisting medicines: A synthesis of qualitative studies of medicine taking. Soc. Sci. Med..

[33] Catz S.L., Kelly J.A., Bogart L.M., Benotsch E.G., McAuliffe T.L. (2000). Patterns, correlates, and barriers to medication adherence among persons prescribed new treatments for HIV disease. Health Psychol..

[34] Horne R., Weinman J. (1999). Patients’ beliefs about prescribed medicines and their role in adherence to treatment in chronic physical illness. J. Psychosom. Res..

[35] Jackson E.S., Tucker C.M., Herman K.C. (2007). Health Value, Perceived Social Support, and Health Self-Efficacy as Factors in a Health-Promoting Lifestyle. J. Am. Coll. Health.

[36] Gallant M.P. (2003). The influence of social support on chronic illness self-management: A review and directions for research. Health Educ. Behav..

[37] Weber K., Johnson A., Corrigan M. (2004). Communcating emotional support and its relationship to feelings of being understood, trust, and self-disclosure. Commun. Res. Rep..

[38] Glasgow R.E., Strycker L.A., Toobert D.J., Eakin E. (2000). A social–ecologic approach to assessing support for disease self-management: The Chronic Illness Resources Survey. J. Behav. Med..

[39] Rempel J.K., Holmes J.G., Zanna M.P. (1985). Trust in close relationships. J. Personal. Soc. Psychol..

[40] Shumaker S.A., Brownell A. (1984). Toward a theory of social support: Closing conceptual gaps. J. Soc. Issues.

[41] Hether H.J., Murphy S.T., Valente T.W. (2014). It’s better to give than to receive: The role of social support, trust, and participation on health-related social networking sites. J. Health Commun..

[42] Macabasco-O’Connell A., DeWalt D., Broucksou K., Hawk V., Baker D., Schillinger D., Ruo B., Bibbins-Domingo K., Holmes G., Erman B. (2011). Relationship Between Literacy, Knowledge, Self-Care Behaviors, and Heart Failure-Related Quality of Life Among Patients With Heart Failure. J. Gen. Intern. Med..

[43] Zhou Y., Kankanhalli A., Yang Z., Lei J. (2017). Expectations of patient-centred care: Investigating IS-related and other antecedents. Inf. Manag..

[44] Ommen O., Janssen C., Neugebauer E., Bouillon B., Rehm K., Rangger C., Erli H.J., Pfaff H. (2008). Trust, social support and patient type—Associations between patients perceived trust, supportive communication and patients preferences in regard to paternalism, clarification and participation of severely injured patients. Patient Educ. Couns..

[45] McDonald H.P., Garg A.X., Haynes R.B. (2002). Interventions to enhance patient adherence to medication prescriptions: Scientific review. JAMA.

[46] Sarkar U., Fisher L., Schillinger D. (2006). Is self-efficacy associated with diabetes self-management across race/ethnicity and health literacy?. Diabetes Care.

[47] Williams D.M., Rhodes R.E. (2016). The confounded self-efficacy construct: Conceptual analysis and recommendations for future research. Health Psychol. Rev..

[48] Conner M., Norman P. (2005). Predicting Health Behaviour: Research and Practice with Social Cognition Models.

[49] Chen A.M.H., Yehle K.S., Albert N.M., Ferraro K.F., Mason H.L., Murawski M.M., Plake K.S. (2014). Relationships between health literacy and heart failure knowledge, self-efficacy, and self-care adherence. Res. Soc. Adm. Pharm..

[50] Fiscella K., Meldrum S., Franks P., Shields C.G., Duberstein P., McDaniel S.H., Epstein R.M. (2004). Patient trust: Is it related to patient-centered behavior of primary care physicians?. Med. Care.

[51] Hall M.A., Dugan E., Zheng B., Mishra A.K. (2001). Trust in Physicians and Medical Institutions: What Is It, Can It Be Measured, and Does It Matter?. Milbank Q..

[52] Lee Y.-Y., Lin J.L. (2009). Trust but verify: The interactive effects of trust and autonomy preferences on health outcomes. Health Care Anal..

[53] Thom D.H., Hall M.A., Pawlson L.G. (2004). Measuring patients’ trust in physicians when assessing quality of care. Health Aff..

[54] Imai H., Furukawa T.A., Hayashi S.-U., Goto A., Izumi K., Hayashino Y., Noda M. (2017). Risk perception, self-efficacy, trust for physician, depression, and behavior modification in diabetic patients. J. Health Psychol..

[55] Islam M.K., Merlo J., Kawachi I., Lindström M., Gerdtham U.-G. (2006). Social capital and health: Does egalitarianism matter? A literature review. Int. J. Equity Health.

[56] Kawachi I., Kennedy B.P., Glass R. (1999). Social capital and self-rated health: A contextual analysis. Am. J. Public Health.

[57] Liang T.-P., Ho Y.-T., Li Y.-W., Turban E. (2011). What Drives Social Commerce: The Role of Social Support and Relationship Quality. Int. J. Electron. Commer..

[58] Karademas E.C. (2006). Self-efficacy, social support and well-being: The mediating role of optimism. Personal. Individ. Differ..

[59] Chow W.S., Chan L.S. (2008). Social network, social trust and shared goals in organizational knowledge sharing. Inf. Manag..

[60] Fernandez S., Chaplin W., Schoenthaler A.M., Ogedegbe G. (2008). Revision and validation of the medication adherence self-efficacy scale (MASES) in hypertensive African Americans. J. Behav. Med..

[61] Hair J.F., Hult G.T.M., Ringle C.M., Sarstedt M. (2017). A Primer on Partial Least Squares Structural Equation Modeling (PLS-SEM).

[62] Fornell C., Larcker D.F. (1981). Evaluating Structural Equation Models with Unobservable Variables and Measurement Error. J. Mark. Res..

[63] Sarstedt M., Henseler J., Ringle C.M., Sarstedt M., Schwaiger M., Taylor C.R. (2011). Multigroup Analysis in Partial Least Squares (PLS) Path Modeling: Alternative Methods and Empirical Results. Measurement and Research Methods in International Marketing (Advances in International Marketing).

[64] Lam W.Y., Fresco P. (2015). Medication adherence measures: An overview. BioMed Res. Int..

[65] Gerteis M., Edgman-Levitan S., Daley J., Delbanco T.L. (1993). Through the Patient’s Eyes: Understanding and Promoting Patient-Centered Care.

[66] Spanjol J., Cui A.S., Nakata C., Sharp L.K., Crawford S.Y., Xiao Y., Watson-Manheim M.B. (2015). Co-production of prolonged, complex, and negative services: An examination of medication adherence in chronically ill individuals. J. Serv. Res..

[67] Thiede M. (2005). Information and access to health care: Is there a role for trust?. Soc. Sci. Med..

